# Bioactive PEEK: Surface Enrichment of Vitronectin-Derived Adhesive Peptides

**DOI:** 10.3390/biom13020246

**Published:** 2023-01-28

**Authors:** Leonardo Cassari, Annj Zamuner, Grazia M. L. Messina, Martina Marsotto, Hongyi Chen, Giovanni Gonnella, Trevor Coward, Chiara Battocchio, Jie Huang, Giovanna Iucci, Giovanni Marletta, Lucy Di Silvio, Monica Dettin

**Affiliations:** 1 Department of Industrial Engineering, University of Padova, Via Marzolo 9, 35131 Padova, Italy; 2 Department of Civil, Environmental, and Architectural Engineering, University of Padova, Via Marzolo 9, 35131 Padova, Italy; 3 Laboratory for Molecular Surface and Nanotechnology (LAMSUN), Department of Chemical Sciences, University of Catania and CSGI, Viale A. Doria, 6, 95125 Catania, Italy; 4 Department of Science, Roma Tre University, Via della Vasca Navale 79, 00146 Roma, Italy; 5 Department of Mechanical Engineering, University College London, London WC1E 6BT, UK; 6 Faculty of Dentistry, Oral & Craniofacial Sciences, King’s College London, London SE1 9RT, UK

**Keywords:** PEEK, surface functionalization, Vitronectin peptides, human osteoblasts, 3D-printing, bone implant

## Abstract

Polyetheretherketone (PEEK) is a thermoplastic polymer that has been recently employed for bone tissue engineering as a result of its biocompatibility and mechanical properties being comparable to human bone. PEEK, however, is a bio-inert material and, when implanted, does not interact with the host tissues, resulting in poor integration. In this work, the surfaces of 3D-printed PEEK disks were functionalized with: (i) an adhesive peptide reproducing [351–359] h-Vitronectin sequence (HVP) and (ii) HVP retro-inverted dimer (D2HVP), that combines the bioactivity of the native sequence (HVP) with the stability toward proteolytic degradation. Both sequences were designed to be anchored to the polymer surface through specific covalent bonds via oxime chemistry. All functionalized PEEK samples were characterized by Water Contact Angle (WCA) measurements, Atomic Force Microscopy (AFM), and X-ray Photoelectron Spectroscopy (XPS) to confirm the peptide enrichment. The biological results showed that both peptides were able to increase cell proliferation at 3 and 21 days. D2HVP functionalized PEEK resulted in an enhanced proliferation across all time points investigated with higher calcium deposition and more elongated cell morphology.

## 1. Introduction

The increase in life expectancy in Western countries has resulted in a higher incidence of bone and cartilage pathologies. Consequently, the demand for, and optimization of orthopedic implants, has grown [1]. For many years, traditional implants have been metallic, usually composed of titanium or titanium alloys due to their resistance to oxidation, excellent mechanical properties, and biocompatibility [2]. The misalignment between the elastic modulus of these metals and that of the cortical bone, together with the release of metal ions from the implant surface, can create associated problems and, in some cases, implant failure. Additionally, polymeric materials are usually not able to withstand repetitive loads without incurring plastic deformations [3]. An exception to this is represented by poly (ether ether ketone) (PEEK). PEEK is a semi-crystalline polyaromatic linear polymer with (–C_6_H_4_–O–C_6_H_4_–O–C_6_H_4_–CO–)_n_ chemical formula, and it possesses incredible mechanical properties. Young’s modulus of PEEK is around 3.6 GPa, similar to the value of cortical bone. It is much lower than any metallic material employed in the common bone implant (110–180 GPa) [4,5]. For this reason, by the late 1990s, PEEK was proposed as an alternative to the metal components of orthopedic implants. More specifically, it is used for femoral prostheses and hip joints [6]. Currently, even dentists are looking at PEEK with increasing interest considering its exceptional mechanical and aesthetic properties [7].

PEEK Young’s modulus is manageable through additive inclusion: for example, by adding carbon reinforcement in a PEEK matrix, its modulus increases up to 18 GPa, which is superimposable to the values of the cortical bone (18.4–20.7 GPa) [8]. Therefore, PEEK implants decrease the effect of stress shielding compared to traditional metal implants. PEEK is a thermoplastic polymer, and it melts at a relatively high temperature (343 °C) compared to most of this class of polymers [8,9]. In the range of its melting temperature, it can be processed using 3D printing. The possibility of employing PEEK polymer in the 3D printing process could increase scientific interest and its future potential [9,10]. Additionally, PEEK has interesting biological properties: it is highly resistant to thermal degradation, to the attack by organic and aqueous environments, and also to biodegradation [11]. Moreover, it is insoluble at room temperature in all conventional solvents, and its thermal properties make it stable in the human body. Therefore, PEEK implants are biocompatible but biologically inert [12]: their applications cause only mild inflammatory responses, and even their debris (that might be produced after a long period of implantation) may give rise to normal reactions of the biological tissue to a foreign body as the worst scenario [13]. Despite being a non-toxic, non-mutagenic, and non-inflammatory material, PEEK remains a bio-inert material. Furthermore, this polymer has a hydrophobic surface, which neither allows protein absorption nor promotes cell adhesion [14].

The growth of new bone tissue in direct apposition with the implant is desirable for all prostheses but becomes a necessity when the osseointegration process is critical, i.e., for elderly patients or patients suffering from multiple pathologies. With this in mind, modification of the implant surface to enhance the adhesion and proliferation of osteoblasts is a favorable approach. Taking this into consideration, enhancing the bioactivity of PEEK presents a desirable challenge to increase the potential benefit from the wide advantages of this material [15].

To date, a number of physical methods for PEEK surface modification have been proposed, such as thermal spraying, pulsed laser, ion sputtering, sandblasting, and electrochemical methods, which require a lot of optimization work and complex equipment. Our strategy concerns the chemical functionalization of PEEK surfaces with bioactive motifs. Previous studies have used the reduction of the carbonyl groups of PEEK to alcohol groups (PEEK-OH). PEEK-OH is then used to obtain isocyanate groups or amino groups on the surface [16,17,18] to be reacted with signal sequences for cell adhesion or proliferation.

In our grafting strategy, the carbonyl groups already present on the PEEK surface react with an amino-oxy group ad hoc inserted in peptide sequence: the method allows the covalent anchoring of bioactive peptides in a single-step without the use of condensing agents or organic solvents [19,20]. This study reports the chemical, physicochemical, mechanical, and biological characterization of PEEK surfaces functionalized with two adhesive peptides: a nonapeptide of human vitronectin (HVP) capable of promoting an osteoblast adhesion mechanism mediated by cell surface glycosaminoglycans [21] and its retro-inversed dimer (D2HVP) which has enhanced biological activity with respect to the first peptide but which is also stabilized against enzymatic degradation [22].

## 2. Materials and Methods

### 2.1. Materials

Acetic acid, acetone, acetonitrile, Fmoc protected Amino acids, Bis-Boc-aminooxy acetic acid (Aoa), Dichloromethane (DCM), N,N-Diisopropylethylamine (DIPEA), N,N-dimethylformamide (DMF), Ethyl cyano(hydroxyimino)acetate (Oxyma Pure), Methanol, Rink-Amide MBHA resin, Triethylamine (TEA), Triethylsilane (TES), 2-(1H-benzotriazol-1-yl)-1,1,3,3-tetramethyluronium hexafluoro phosphate (HBTU) were purchased from Merck Millipore (Burlington, MA, USA). Diethyl ether, Trifluoroacetin acid (TFA) were purchased from Biosolve (Valkenswaard, Holland). 2-Proponal for HPLC (aldehyde and ketone free) was purchased from PanReac AppliChem (Darmstadt, Germany). Ethanol was purchased from Carlo Erba (Milan, Italy). N-Methyl-2-pyrrolidone (NMP) was purchased from Iris Biotech GmbH (Marktredwitz, Germany).

### 2.2. Synthesis of the Peptide Aoa-x-HVP

Aoa-x-HVP (sequence: H-Aoa-(7-aminoheptanoic acid)-Phe-Arg-His-Arg-Asn-Arg-Lys-Gly-Tyr-NH_2_) was synthesized using the Solid Phase Peptide technique (SPPS) on Tink amide MBHA resin by a Syro I synthesizer (Multisyntech, Witten, Germany). All the amino acids were protected with a Fmoc group on the N-termini and with a Pbf, Trt, Boc, or tBu group on the side chain. Every amino acid was introduced through a double coupling with five equivalents of each amino acid, five equivalents of activating agent HBTU/Oxima Pure, and ten equivalents of DIPEA with respect to the resin reactive groups used for each coupling of 45 min. Aoa was added to the sequence through a double coupling with five equivalents of each amino acid, five equivalents of activating agent HATU/Oxima Pure, and ten equivalents of collidine with respect to the resin reactive groups were used for each coupling of 45 min.

After the addition of Aoa, the peptide was cleaved from the resin and deprotected from Bis-Boc of Aoa and the side-chain protecting groups using 4.75 mL TFA, 0.125 mL TES, and 0.125 H_2_O for 1.5 h at room temperature.

The resin was filtered off, and the solution was concentrated and added with cold diethyl ether. The product was precipitated and filtered. The identity of the peptide was determined by mass spectrometry (theoretical mass = 1432.587 Da; experimental mass = 1431.8156 Da; ESI-TOF, Mariner System 5220, Applied Biosystem, PerkinElmer, Foser City, CA, USA). The peptide Aoa-x-HVP was purified by RP-HPLC (purity grade 98.74%) and characterized by analytical RP-HPLC (condition: Jupiter C18 column [5 μm, 300 Å, 4.6 × 205 mm], eluent A: 0.05% TFA in H_2_O; eluent B: 0.05% TFA in 40% isopropanol and 60% MilliQ water; gradient: from 20 to 50% B in 30 min, flow rate: 0.5 mL/min; detector at 214 nm. t_R_ = 13.215 min).

### 2.3. Synthesis of the Peptide Aoa-x-F-HVP

Aoa-x-F-HVP (sequence: H-Aoa-(7-aminoheptanoic acid)-(p-F)Phe-Arg-His-Arg-Asn-Arg-Lys-Gly-Tyr-NH_2_, where (p-F)Phe indicates a para-Fluoro Phenylalanine residue) was synthesized using the Solid Phase Peptide technique (SPPS) on Tink amide MBHA resin by a Syro I synthesizer (Multisyntech, Witten, Germany). All the amino acids were protected with a Fmoc group on the N-termini and with a Pbf, Trt, Boc, or tBu group on the side chain. Every amino acid was introduced through a double coupling with five equivalents of each amino acid, five equivalents of activating agent HBTU/Oxima Pure, and ten equivalents of DIPEA with respect to the resin reactive groups used for each coupling of 45 min. Aoa was added to the sequence through a double coupling with five equivalents of each amino acid, five equivalents of activating agent HATU/Oxima Pure, and ten equivalents of collidine with respect to the resin reactive groups were used for each coupling of 45 min.

After the addition of Aoa, the peptide was cleaved from the resin and deprotected from Bis-Boc of Aoa and the side-chain protecting groups as reported for Aoa-x-HVP.

The resin was filtered off, and the solution was concentrated and added with cold diethyl ether. The product was precipitated and filtered. The identity of the peptide was determined by mass spectrometry (theoretical mass = 1450.58 Da; experimental mass = 1450.32 Da; SCIEX TOF-TOF 4800 instrument). The peptide Aoa-x-F-HVP was purified by RP-HPLC (purity grade 99.36%) and characterized by analytical RP-HPLC (condition: Jupiter C18 column [5 μm, 300 Å, 4.6 × 205 mm], eluent A: 0.05% TFA in H_2_O; eluent B: 0.05% TFA in 40% isopropanol and 60% MilliQ water; gradient: from 20 to 50% B in 30 min, flow rate: 0.5 mL/min; detector at 214 nm. t_R_ = 14.688 min).

### 2.4. Synthesis of the Peptide Aoa-x-D2HVP

Aoa-x-D2HVP (sequence: H-Aoa-(7-aminoheptanoic acid)-(DTyr-Gly-DLys-DArg-DAsn-DArg-DHis-DArg-DPhe)_2_-NH_2_) was synthesized using the Solid Phase Peptide technique (SPPS) on Rink amide MBHA resin by a Syro I synthesizer (Multisyntech, Witten, Germany). All the amino acids are of D- type, and they were protected with a Fmoc group on the N-termini and with a Pbf, Trt, Boc, or tBu group on the side chain. Every amino acid was introduced through a single coupling with five equivalents of each amino acid, five equivalents of activating agent HATU/Oxima Pure, and ten equivalents of DIPEA with respect to the resin reactive groups used for each coupling of 45 min. Aoa was added to the sequence through a double coupling with five equivalents of each amino acid, five equivalents of activating agent HATU/Oxima Pure, and ten equivalents of collidine with respect to the resin reactive groups were used for each coupling of 45 min. 

After the addition of Aoa, the peptide was cleaved from the resin and deprotected from Bis-Boc of Aoa and the side-chain protecting groups as reported for Aoa-x-HVP.

The resin was filtered off, and the solution was concentrated and added with cold diethyl ether. The product was precipitated and filtered. The identity of the peptide was determined by mass spectrometry (theoretical mass = 2647.984 Da; experimental mass = 2647.02 Da; SCIEX TOF-TOF 4800 instrument). The peptide was purified by RP-HPLC (purity grade 95%) and characterized by analytical RP-HPLC (condition: Vydac C18 column [5 μm, 300 Å, 4.6 × 250 mm], eluent A: 0.05% TFA in H_2_O; eluent B: 0.05% TFA in 40% isopropanol and 60% MilliQ water; gradient: from 5 to 45% B in 40 min, flow rate: 0.5 mL/min; detector at 214 nm. t_R_ = 32.510 min).

### 2.5. 3D-Printing of PEEK Scaffold

AutoDesk Fusion 360 (San Rafael, CA, USA) computer-aided-design (CAD) software was used to design PEEK disks 10 mm in diameter and 4 mm in height and exported in an *stl* format into Simplify 3D software (Cincinnati, OH, USA). The files were then sliced to generate printer-ready G-code and printed using an Apium P155 Peek filament printer (Apium Additive Technologies GmBh, Willy, Karlsruhe, Germany). Peek filament (Vitrex Rotherham, Rotherham, UK) 1.75 mm in diameter was extruded through a 0.4 mm diameter nozzle at a temperature of 485 °C onto a print bed with a temperature of 130 °C to create the PEEK disks with a 100% infill at a print speed of 33.3 mm/s. The resulting 3D-printed PEEK disks possess a flat surface and a patterned one.

### 2.6. PEEK Surface Functionalization

PEEK scaffolds were functionalized with all the peptides. The linkages between the amino-oxy group on the N-terminus of the peptide chain and ketonic groups on the PEEK surface are realized by means of chemoselective ligation. Each peptide was dissolved in 40 mM monobasic sodium phosphate at pH 6 and left to react with PEEK samples for 24 h at room temperature. Peptide concentrations used were 10^−5^, 10^−4^, and 10^−3^ M. After that, the disks were rinsed three times with buffer and three times with MilliQ water.

### 2.7. X-ray Photoelectron Spectroscopy (XPS) Measurements

XPS analysis was performed with a homemade instrument, consisting of preparation and analysis of UHV chambers separated by a gate valve. The analysis chamber is equipped with a six-degree-of-freedom manipulator and a 150 mm mean radius hemispherical electron analyzer with a five-lens output system combined with a 16-channel detector giving a total instrument resolution of 1.0 eV as measured at the Ag 3d 5/2 core level. Samples were introduced in the preparation chamber and left outgassing overnight at a base pressure of about 10^−8^ Torr before introduction in the analysis chamber. The typical vacuum pressure in the analysis chamber during measurements was in the 10^−9^–10^−10^ Torr range. The used X-ray radiation is a non-monochromatic Mg Kα(1253.6 eV). The spectra were energy referenced to the C 1s signal of aromatic and aliphatic C atoms having a binding energy BE = 284.7 eV. Curve-fitting analysis of the C1s, N1s, O1s, and F1s spectra was performed using Gaussian profiles as fitting functions after subtraction of a Shirley-type background. Atomic ratio values were calculated from peak intensities. The surface atomic concentrations were determined from photoelectron peak areas using the atomic sensitivity factors reported by Scofield [23]. When several different species were identified in a spectrum, the same FWHM value was set for all individual photoemission bands. XPS analyses were performed on flat PEEK surfaces.

### 2.8. Atomic Force Microscopy (AFM) Analysis

AFM analyses were carried out on different functionalized PEEK disks at room temperature in order to evaluate the effects of the peptides on the mechanical properties of interfaces separately. The Young modulus was calculated from the collected force–distance curves measured with the NTEGRA AFM (NT-MDT, Moscow, Russia). Stiff single-crystal silicon cantilevers with a symmetric tip shape were used (model Tap300Al-G, BudgetSensors, Bulgaria: nom. Frequency 300 kHz, nom. Spring constant 40 Nm^−1^, tip radius < 10 nm). AFM measurements were made in triplicate. The probe was characterized by measuring the cantilever spring constant by the Sader method [24]. Each probe was calibrated by performing a force curve on a hard-cleaned substrate (<100> silicon wafer), where no indentation occurred. Probe calibration was needed to calculate their sensitivity and the spring constant. The Young modulus was obtained from the experimental force–distance curves by the Derjaguin–Müller–Toporov (DMT) model [25] using the following equation:

(1) $$F+F_{ad}=\frac{4E_{S}}{3\left(1-\nu_{s}^{2}\right)}R^{\frac{1}{2}}\delta^{\frac{3}{2}}$$

where *F* is the applied force; *F_ad_* is the adhesion force; *Es* is Young’s modulus; *ν_s_* is the Poisson’s ratio for the sample; *R* is the radius of the spherical indenter; and *δ* is the elastic indentation depth. Each surface was indented in different areas, and about three hundred curves were acquired. The tip-sample approaching rate was set to 0.3 µms^−1^ for all force curves. Young’s modulus was calculated by fitting experimental curves with the DMT model in the elastic region. Elastic modulus distribution was assessed with OriginPro 8.5 software.

For adhesion force measurements, Au-coated silicon nitride tips were used. The spring constants of the cantilevers used were in the range of 0.003−0.13 N/m. Individual spring constants were calibrated using the Sader method. The adhesion force is taken as the pull-off force required to separate the AFM tip from the surface. The surfaces were probed in different areas, and about three hundred approach-retract cycles were acquired for each system. The tip-sample approaching velocity was set to 0.3 μs^−1^ for all force curves. The adhesion force between the tip and surface can be calculated from the deflection distance of the cantilever and the cantilever spring constant by applying Hook’s law:

*F* = *k* × Δ*L* (2)

where *F* is the force (nN), *k* is the spring constant of the cantilever, and Δ*L* is the deflection distance (nm). The cantilever spring constants were equal to 0.05 N/m for PEEK-HVP and 0.08 N/m for PEEK-D2HVP.

### 2.9. Water Contact Angle (WCA) Analysis

The surface wettability was traced by measuring the static water contact angle (WCA). An OCA30 instrument (Dataphysics) was used at 25 °C and 65% relative humidity. Probe liquid drops of 2 µL of volume were dispended on different zones of each sample surface, and by digital image analysis, the static contact angle, as the tangent to the drop at the point of contact with the surface, both on the right and on the left side, has been acquired. At least three measurements were made for each sample and then averaged. WCA analyses were performed on flat PEEK surfaces.

### 2.10. Biological Assays

All biological studies were performed on the patterned surface of the 3D-printed PEEK disks.

#### 2.10.1. Cell Culture

Human osteoblasts (HOB) were used to evaluate the functionalized PEEK disks. Prior to seeding, PEEK specimens were sterilized in 70% ethanol for 24 h and then washed with PBS. Cells were cultured in Dulbecco’s Modified Eagle Medium (DMEM) containing 10 mL of 1 M HEPES, 5 mL of 200 mM L-glutamine, 5 mL of 100× penicillin/streptomycin, 50 mL of Fetal Calf Serum (FCS), 5 mL of minimal essential medium and 0.075 g of solid ascorbate acid. HOB cells were microseeded onto the surfaces of the disks at a density of 1 × 10^5^ cells per disc. Seeded samples were incubated at 37 °C and 5% CO_2_ for the time duration required or all tests performed. For gene expression evaluation, a density of 2 × 10^5^ HOB cells/per disc was used. Culture media was changed every three days for optimal growth conditions.

#### 2.10.2. Live and Dead

Cell viability was assessed using a LIVE/DEAD^TM^ Viability/Cytotoxicity Kit (Thermo Fisher Scientific, Waltham, MA, USA) after 48 h of cell culture. Two PBS washings were performed prior to exposure to live/dead stains. Images were captured with an Olympus IX51 fluorescent microscope. ImageJ software was used to display and merge images of living cells (green fluorescence at 530 nm) and dead cells (red fluorescence at 600 nm) of the same location into one image.

#### 2.10.3. Scanning Electron Microscopy (SEM) Analysis

The morphology of HOB cells on the 3D-printed PEEK structures was observed using SEM (Zeiss Gemini SEM 360, Jena, Germany) at a voltage of 10kV. Forty-eight hours after seeding, samples were fixed with 2.5% glutaraldehyde in 0.1 M sodium cacodylate buffer for at least 24 h at 4 °C to preserve the structure of cells. Following this, the glutaraldehyde was removed, and the samples were rinsed with 0.1 M sodium cacodylate buffer solution before treatment with 1% osmium tetroxide in 0.1 M sodium cacodylate buffer for 1 h. The osmium tetroxide was then removed, and samples were further rinsed with 0.1 M sodium cacodylate buffer. 1% tannic acid in 0.1 M sodium cacodylate buffer was used to treat the samples for 1 h. Following this, the samples were dehydrated in a series of aqueous ethanol solutions (20%, 30%, 40%, 50%, 60%, 70%, 90%, 96%, and 100%). For each concentration, the samples were treated twice, and each treatment was 5 min. After dehydration, the samples were air-dried and sputter coated with gold prior to observation under SEM.

#### 2.10.4. Alizarin Red

Calcium deposition was evaluated by Alizarin Red S staining on HOB cells. Alizarin red S is an anthraquinone dye that reacts with calcium and forms an Alizarin Red S/calcium complex that is birefringent. HOB cells were cultured for 21 days, and the disks were then washed in PBS, fixed with 10% formal saline for 15 min, and washed with deionized water three times. 2% Alizarin red S solution was added to the specimens for 15 min, followed by five washes in 50% ethanol. For qualitative assessment, images of the stained scaffolds were taken. A quantitative assessment was conducted using Lee et al. protocol [26]. The bound stain taken up by the cells on the disks was released using 1 mL of 10% cetylpyridium chloride (CPC) solution added to each well overnight (24 well plates). Aliquots (100 μL) of the supernatant were removed and read using a microplate reader (Opsys MRTM 96-well microplate reader, Dynex Technologies, Chantilly, VA, USA) at 570 nm. A standard curve, using known concentrations of alizarin red S in CPC solution, was used for the quantification. Cell mineralization results were expressed as μmol of calcium per well as 1 mole of Alizarin red S binds to 2 mol of calcium in an Alizarin red S-calcium complex [27].

#### 2.10.5. Proliferation Assay

The proliferation of osteoblasts cultured on functionalized and non-functionalized PEEK disks was assessed at 1, 4, 7, 14, or 21 days using the AlamarBlue^TM^ kit. For each sample, three replicates were measured with a microplate reader (Opsys MRTM 96-well microplate reader, Dynex Technologies, Chantilly, VA, USA), which measured the fluorescence values at 570 nm test wavelength and 630 nm reference wavelength.

#### 2.10.6. Quantitative Real-Time Polymerase Chain Reaction (qRT-PCR)

Specific mRNA transcript levels coding human SRY-Box transcription factor 9 (Sox9), Runt-related transcription factor 2 (Runx2), Alkaline Phosphatase (Alp), and Osteocalcin (Oct) were quantified in osteoblast cells cultured for 24 h on functionalized and non-functionalized PEEK. At the end of incubation, the total RNA was extracted using the SV Total RNA Isolation System kit (Promega, Milan, Italy). Contaminating DNA was removed by DNase I digestion (Omega Bio-Tek, Norcross, GA, USA). cDNA synthesis and subsequent polymerization were performed in one step using the iTaq Universal SYBR Green One-Step Kit (Bio-Rad, Hercules, CA, USA). The reaction mixture contained a 200 nM forward primer, 200 nM reverse primer, iTaq universal SyBR Green reaction mix, iScript reverse transcriptase, and a 200 ng total RNA. Real-time PCR was performed using the ABI PRISM 7700 Sequence Detection System (Applied Biosystems, Milan, Italy). Data were quantified by the ∆∆CT method using human Gapdh as the reference gene. Target and reference genes were amplified with efficiencies near 100%. The oligonucleotides used for PCR are listed in Table 1.

#### 2.10.7. Statistical Analysis

All assays were performed at least in triplicate, and all values are reported as mean ± standard deviation.

Data were analyzed and processed with Prism (GraphPad Software 9). Alizarin Red S results were compared by using a one-way analysis of variance (ANOVA) test. Tukey’s multiple comparisons test was carried out to confront every condition. AlamarBlue^TM^ and gene expression results were compared with two-way ANOVA tests, followed by Tukey’s post-hoc test.

The significance level applied was 5%.

## 3. Results

### 3.1. Determination of the Best Functionalizing Conditions

PEEK disks were functionalized with Aoa-x-F-HVP to allow direct individuation of the surface peptide through XPS fluorine atom detection.

The F/C ratio is directly proportional to the quantity of peptide on the surface of the sample: the data demonstrate that, varying the concentration of peptide solution (from 10^−5^ M to 10^−3^ M), the surface peptide presence increases (Table 2).

Proliferation on PEEK disks functionalized with Aoa-x-HVP at 10^−5^ M, 10^−4^ M, and 10^−3^ M was measured using the AlamarBlue^TM^ kit. This assay was performed to assess the responsiveness of cells to different concentrations of peptides used to functionalize the PEEK scaffolds. As a control, untreated PEEK scaffolds were used.

**Figure 1:**
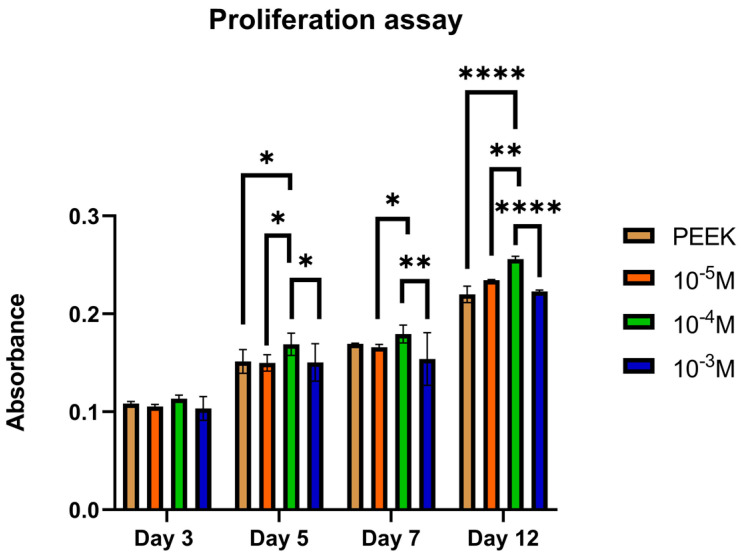
HOB proliferation assay. Quantification of cell proliferation at 3, 5, 7, and 12 days of cells seeded on PEEK samples functionalized with different concentrations of Aoa-x-HVP (10^−3^ M, 10^−4^ M, 10^−5^ M). Values are reported as mean ± SD. * *p* < 0.05, ** *p* < 0.01, **** *p* < 0.0001, *n* = 3 per sample.

Figure 1 shows the different metabolic activities of HOB cells on the scaffolds, which consistently increased during 12 days of culture (cells were actively proliferating on the samples). More in particular, from day 5 onwards, 10^−4^ M scaffolds outperformed the other groups by significantly increasing the HOB cells proliferation rate, with an average absorbance peak of 0.256 at day 12. This concentration was selected for functionalizing PEEK disks in the next experiments.

**Figure 2:**
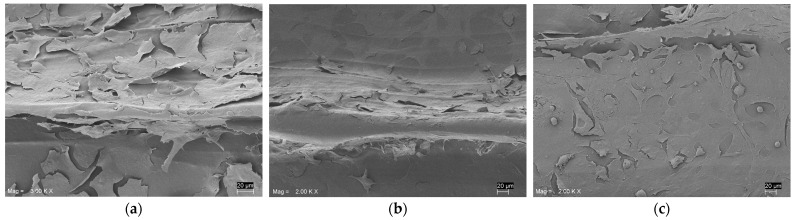
SEM of HOB cells seeded for 24 h on control. (**a**) Unfunctionalized PEEK sample, (**b**) PEEK-HVP, and (**c**) PEEK-D2HVP. Cells were seen to adhere on both surfaces; in the non-functionalized PEEK, cells were elongated and appeared flattened in the functionalized PEEK scaffolds. Cells had formed layers covering the entire surface, and some rounded dividing cells were also visible. Scale bars = 20 μm.

Cellular adhesion and morphology of HOB cells on the functionalized scaffolds were observed by SEM (Figure 2).

### 3.2. Surface Characterization

The characterization was carried out on the flat surfaces of PEEK disks functionalized with 10^−4^ M peptide concentration solution of Aoa-x-HVP or Aoa-x-D2HVP and named respectively PEEK-HVP and PEEK-D2HVP.

#### 3.2.1. XPS Analysis

**Figure 3:**
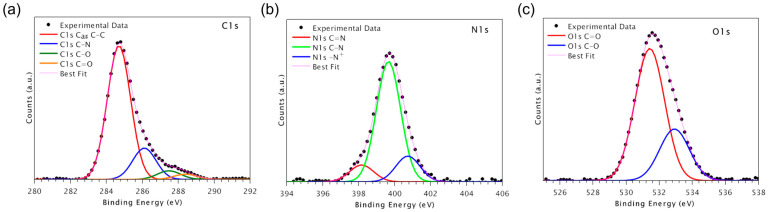
XPS spectra and peak fit results of sample PEEK-D2HVP. C1s (**a**), N1s (**b**), and O1s (**c**) spectra of PEEK-D2HVP samples.

For all samples, XPS measurements were carried out at the C1s, N1s, and O1s core levels. XPS data (BE, FWHM, atomic ratios) are reported in Table S1 in Supplementary Materials; measured N/C atomic ratios are reported in Table 3. C1s, N1s, and O1s core level spectra of sample PEEK-D2HVP are shown in Figure 3 (for completeness, the spectra of PEEK and PEEK-HVP are reported in Figure S1 of Supplementary Materials).

C1s spectra (Figure 3a) are made of four components. The first component, fixed at 284.7 eV, is assigned to aromatic and aliphatic carbons; the second at around 286.0 eV to C-N; the third at about 287.6 eV is due to C-O carbons; the last at about 288.5 eV to C=O carbons [28]. 

N1s spectra (Figure 3b) are made of three components. The first at around 398.3 eV is due to C=N nitrogens of Arg and His; the second at 399.7 eV to C-N nitrogens of the peptide backbone; the third at around 401.5 to protonated nitrogens [29].

O1s spectra (Figure 3c) are made of two components. The first component, at nearly 531.5 eV, is associated with C=O oxygens; the second, at nearly 533.2 eV, with C-O oxygens.

Peptide immobilization on the PEEK surface is evidenced by the appearance of the N1s signals typical of the peptide; the amount of surface peptide is similar in the two samples.

#### 3.2.2. Atomic Force Microscopy (AFM) Analyses

**Figure 4:**
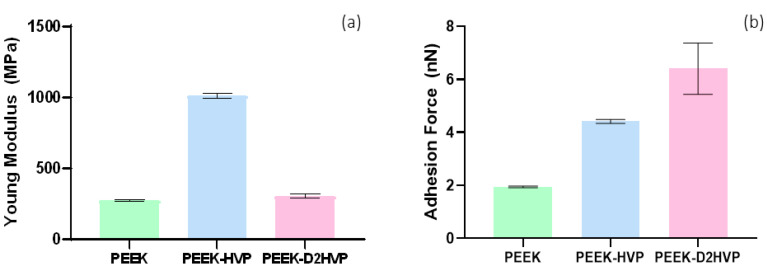
AFM analysis of functionalized PEEK. (**a**) Surface Young’s modulus and (**b**) adhesion forces for the bare and functionalized PEEK. Values are reported as mean ± SD. *n* = 3.

As shown in Figure 4a, both functionalized PEEK samples presented an increased stiffness denoted by a higher value of the surface Young’s modulus. The effect is particularly evident in the case of PEEK-HVP, where an almost quadruplicate value with respect to the control has been found. The increase of surface rigidity with the functionalization of the PEEK substrate may be explained in terms of the expected stronger interchain interactions and, thus, with the consequent increase in the value of Young’s modulus for all the analyzed surfaces. In particular, the effect is enhanced for PEEK functionalized with HVP, as far as the shorter peptide sequence can interlock more easily within the polymer chains.

Figure 4b shows the adhesion forces measured on the bare and peptide-functionalized surfaces. Both PEEK-HVP and PEEK-D2HVP show enhanced adhesion tip-sample forces, compared to non-functionalized PEEK, since the peptide at the interface promotes intermolecular interactions with the tip. Again, as we previously noted, the HVP peptide is suggested to intercalate in the polymer chains so that its possible to interact with the tip that is “hidden” with respect to the peptide D2HVP, which, bearing a double chain, has a wider area of interaction with the AFM tip, resulting in a higher adhesion force.

#### 3.2.3. Water Contact Angle (WCA)

**Figure 5:**
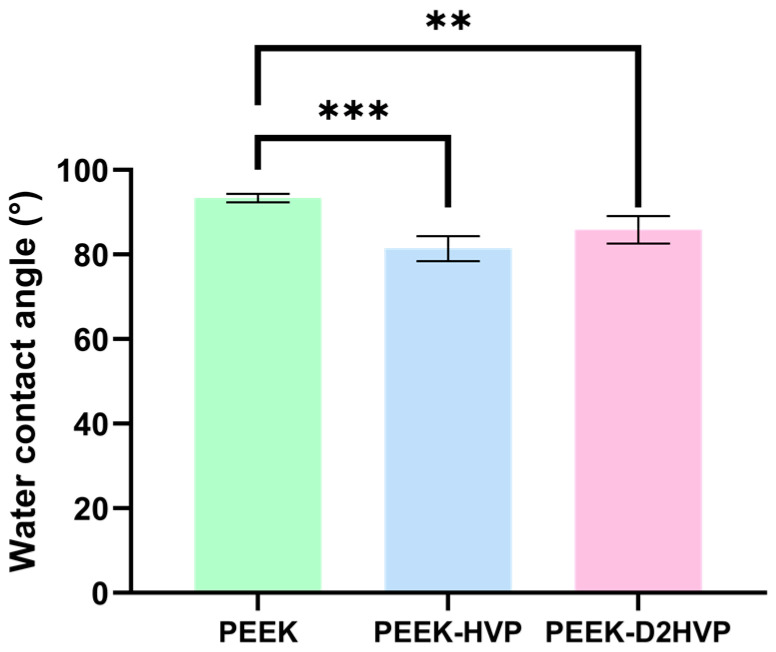
WCA analysis on bare and functionalized PEEK. Values are reported as mean ± SD. ** *p*-value < 0.01 and *** *p*-value < 0.001. *n* = 3.

Both peptides on PEEK disks slightly increase the seeming hydrophilicity of the polymer surface (*i.e.*, the slight decrease of the static water contact angle, Figure 5). The substantial similarity of the WCA values should be related to the fact that essentially the same amino acids are exposed at the surfaces, thus smoothing, together with the rough nature of the surfaces, the wetting behavior of the functionalized PEEK surfaces.

The mean values of the water contact angle measured on the different samples are reported in Table 4.

### 3.3. Biological Tests

#### 3.3.1. Live and Dead

**Figure 6:**
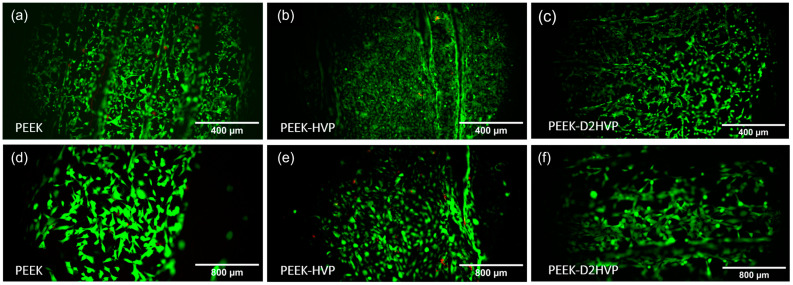
Live and Dead staining of HOB cultured for 48 h on non-functionalized and functionalized PEEK at 48 h. A large number of viable (green) cells e were observed, with very few dead (red) cells seen in all images (**a**–**f**). A greater number of rounded cells were observed on PEEK-HVP (**b**,**e**).

As observed in Figure 6, HOB colonized all test samples. Generally, no cell death (red cells) was observed on test surfaces after 48 h in culture, demonstrating that there was no cytotoxic effect. Only PEEK-HVP showed a few dead cells (Figure 6b,e). The morphology of seeded cells appeared elongated on both PEEK (Figure 6a,d) and PEEK-D2HVP (Figure 6c,f) samples, with a more rounded morphology observed on PEEK-HVP.

#### 3.3.2. Alizarin Red

**Figure 7:**
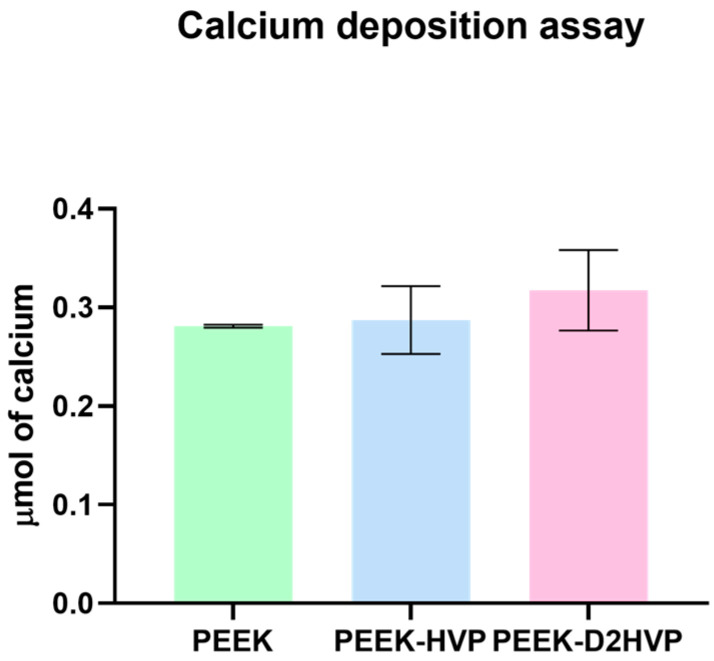
Mineralization assay. Evaluation of calcium deposition on differently functionalized PEEK disks at 21 days.

As reported in Figure 7, both PEEK-HVP and PEEK-D2HVP increased the deposition of calcium ions after 21 days in culture, although no statistical significance was detected.

#### 3.3.3. Proliferation Assay

**Figure 8:**
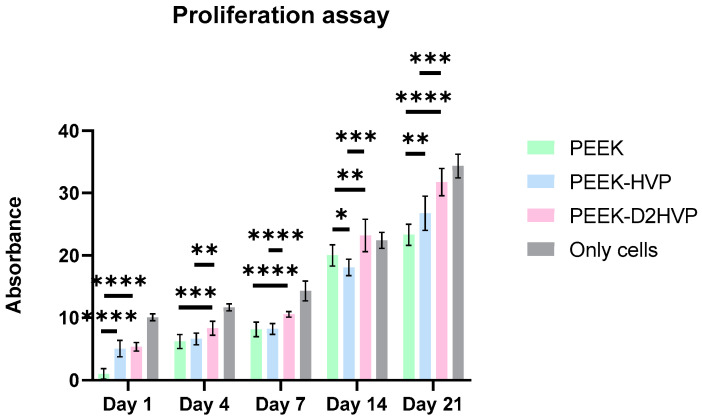
HOB proliferation assay. Results were achieved at 1, 4, 7, 14, and 21 days from cell seeding. * *p*-value < 0.05, ** *p*-value < 0.01, *** *p*-value < 0.001, and **** *p*-value < 0.0001.

HOB proliferation on differently functionalized PEEK samples was evaluated at 1, 3, 7, 14, and 21 days from cell seeding using the AlamarBlue^TM^ kit (Figure 8). Both PEEK-HVP and PEEK-D2HVP showed a significant increase in cell proliferation on days 1 and 21. The functionalization with D2HVP peptide gave the best results at all time points.

#### 3.3.4. Gene Expression

**Figure 9:**
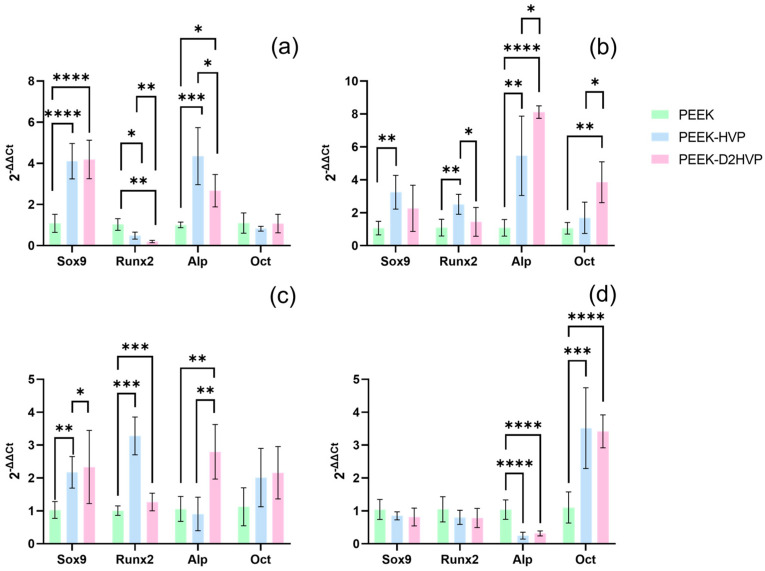
qRT-PCR assay. Gene expression of Sox9, Runx2, Alp, and Oct, was evaluated on differently functionalized PEEK samples at 1 day (**a**), 7 days (**b**), 14 days (**c**), and 21 days (**d**) from HOB seeding. * *p*-value < 0.05, ** *p*-value < 0.01, *** *p*-value < 0.001, and **** *p*-value < 0.0001.

Figure 9a shows that the Sox9 gene was up-regulated at day 1 by both the functionalized samples compared to the control (Figure 9a). At day 7 and day 14 (Figure 9b,c), only functionalization with HVP-induced over-expression. Regarding the factors related to the cell differentiation into osteoblasts, Runx2 presents a statistically significant down-regulation on day 1 and a significant up-regulation on day 14 for both functionalized surfaces (Figure 9). The graphs about Alp and Oct expression (that are respectively early and late mineralization activity markers) have opposite dynamics: Alp was up-regulated in the first time points by the presence of peptides, and then it decreased at the late days, while Oct showed an increasing trend up to the greatest significant improvements in the gene expression at day 21 (Figure 9d) for peptide-enriched surfaces with respect to control.

## 4. Discussion

We investigated the effect of functionalizing PEEK to determine whether the performance could be enhanced. Preliminarily, experiments were performed to ascertain whether the use of increasing concentrations of peptides led to different surface peptide densities. For this purpose, an analog of the HVP peptide was prepared by substitution of Phe → (p-F) Phe. The presence of fluorine as a marker allowed for easier XPS analysis. From the XPS data, it can be deduced that the increase in the concentration of peptide in solution leads to an increased surface peptide density, as evidenced by the enhancement of the F/C ratio when passing from the operating concentration of 10^−5^ M up to the concentration 10^−3^ M. Further investigations examined cell proliferation on variable surface peptide densities. The data obtained indicated that a concentration of 10^−4^ M HVP functionalization solution was able to promote the proliferation of human osteoblast cells at all times investigated.

The advantage of the anchoring method proposed via oxime is that it is extremely simple and absolutely free of unwanted reactions or side effects (persistence of condensing agents or traces of organic solvents). We had evidence of the chemistry solidity of this anchoring strategy in chemoselective conjugations where the conjugate identity determination was possible by MALDI-TOF spectrometry [30]. Furthermore, the anchoring strategy via imine was performed in the functionalization of Mn-containing Bioactive Glass-Ceramics with a BMP-2 fragment. In this case, the band of C-N stretching mode of the secondary amine formed upon the reduction of the Schiff’s base was detected [31]. Compared to the Schiff base anchoring already proposed by Becker M. et al. [32] for PEEK functionalization, the anchoring via oxime does not use an easily reversible reaction, so it does not require the reduction of the Schiff base to amine. The dynamic co-assembly, in fact [33], is not functional to obtain biomaterials that encourage cell adhesion as it is the stable anchoring of the adhesive sequence to the material that mediates cell adhesion. In contrast, the release of the adhesive sequence from the surface of the material can induce adhesion inhibition as this, as a soluble molecule, blocks cell integrins [34].

The enrichment of the surface with adhesive peptides was ascertained with XPS analysis. From the N/C ratios, it is clear how the two peptides reacted differently: considering that D2HVP has a double sequence then HVP (double nitrogen content), it can be estimated that the surface concentration of D2HVP is about 1/3 compared to that obtained using the peptide HVP.

The presence of peptides modified the surface properties of the material, making PEEK more hydrophilic and, therefore, more suitable for interaction with cells. The evaluation of Young’s modulus by AFM showed that functionalization of the surface increases its stiffness. The result is particularly relevant following the functionalization with the shorter HVP peptide. The hypothesis is that this smaller peptide compared to D2HVP, is able to fill the gaps between the surface polymer chains while its retro-inverted dimer does so only in part due to the greater steric hindrance. Both peptides increased the adhesion forces with the AFM cantilever compared to the control. In particular, the presence of the HVP peptide doubles the adhesive force with the cantilever tip compared to PEEK alone, while the D2HVP peptide tripled the adhesion force compared to the control. This may be due to the longer sequence of the retro-inverted dimer compared to HVP.

The Live and Dead staining at 48 h showed a large number of viable cells attached to all the surfaces, and only a few red dead cells were visible on the HVP sample but were absent on the D2HVP sample. The elongated morphology, index of spreading, and, therefore, adhesion were observed on the control PEEK, and the PEEK was functionalized with D2HVP. Cells on PEEK-HVP appeared more rounded, indicating more metabolically active cells.

Quantification of the cells at different time points was assessed with the AlamarBlue^TM^ test. On day one, an increase in the number of adhered cells was observed on both functionalized surfaces when compared to the control by about 300% in accordance with the adhesive nature of the sequences [22]. At the subsequent time points (4, 7, and 14 days), the surface functionalized with the D2HVP peptide induced a greater proliferation than the control. At 21 days post-seeding, both functionalized surfaces had a significantly higher number of cells than the control. This confirms that f, the D2HVP peptide, resulted in an increase in proliferation rate. The PEEK functionalization with the D2HVP peptide produced an enhanced proliferation rate at all time points investigated. The best biological response was observed on samples with D2HVP. This could be attributable to the greater stability of the sequence against serum proteases [22].

The calcium ion deposition was greater on the D2HVP peptide, indicating an increase in mineralization with respect to its monomer and control: the difference, however, was not significant. Furthermore, HVP and D2HVP functionalization significantly enhanced the gene expression of Sox9 at one day from cell seeding, and although it induced a down-regulation of Runx2 at one day, this is inverted at days 7 and 14, suggesting a positive trend over time (for HVP only). Expression of Sox9 has been observed in in vitro osteogenic differentiation of human Mesenchymal Stem Cells (hMSCs) [35]. However, the enhanced expression seen in this study could be indicative of a possible role for Sox9 in mature osteoblasts in direct osteogenesis, supported by overexpression of ALP at early stages and of Oct at later stages in functionalized samples; this interesting observation warrants further investigation.

Scaffolds play a significant role in cell adherence, a major contributing factor to subsequent morphology, proliferation, and maturation of cells. The presence of bioactive sequences on the PEEK surface allowed the design of an engineered surface able to promote the proliferation and differentiation of human osteoblasts. Results have shown an increase in adherence, proliferation, and maturation of osteoblasts. To further examine and elucidate the mechanistic actions of these peptides, future studies will explore their role in the induction of stem cells towards the osteogenic lineage and examine regulatory signaling pathways in vitro.

## Figures and Tables

**Table 1:** Oligonucleotide sequences of the genes evaluated by qRT-PCR.

Gene	Sequence	
Gapdh	fw: 5′-acagttgccatgtagacc-3′	rv: 5′-ttgagcacagggtacttta-3′
Sox9	fw: 5′-aggaagctcgcggaccagtac-3′	rv: 5′-ggtggtccttcttgtgctgcac-3′
Runx2	fw: 5′-cccagtatgagagtaggtgtcc-3′	rv: 5′-gggtaagactggtcataggacc-3′
Alp	fw: 5′-caacgaggtcatctccgtgatg-3′	rv: 5′-taccagttgcggttcaccgtgt-3′
Oct	fw: 5′-cgctacctgtatcaatggctgg-3′	rv: 5′-ctcctgaaagccgatgtggtca-3′

**Table 2:** XPS evaluation.

Sample	Signal	Assignment	Atomic Ratios (%)
PEEK	F1s	F/C	0.2
PEEK-F-HVP 10^−5^ M	F1s	F/C	0.2
PEEK-F-HVP 10^−4^ M	F1s	F/C	0.3
PEEK-F-HVP 10^−3^ M	F1s	F/C	0.4

**Table 3:** XPS analysis. Quantification of N/C ratio for each functionalized scaffold (PEEK-HVP and PEEK-D2HVP) and not functionalized scaffold (PEEK).

Sample	N/C
PEEK	/
PEEK-HVP	0.12
PEEK-D2HVP	0.089

**Table 4:** The water contact angle of functionalized and unfunctionalized PEEK samples.

Sample	Contact Angle ± Standard Deviation
PEEK	93.41 ± 0.83
PEEK-HVP	81.41 ± 2.69
PEEK-D2HVP	85.90 ± 3.00

## Data Availability

Not applicable.

## Supplementary Materials

The following supporting information can be downloaded at: https://www.mdpi.com/article/10.3390/biom13020246/s1, Table S1: XPS results; Figure S1: XPS spectra and peak fit results of sample PEEK and PEEK-HVP.
