# Supplementary material for: Bioactive PEEK: Surface Enrichment of Vitronectin-Derived Adhesive Peptides
Source: Biomolecules. 2023 Jan 28;13(2):246. doi: 10.3390/biom13020246 (PMC9953662; doi:10.3390/biom13020246)
Supplement: Supplementary file 1 [file biomolecules-13-00246-s001.zip › biomolecules-2067901-supplementary.pdf]

biomolecules

MDPI

Supplementary Materials

Article

Bioactive PEEK: Surface Enrichment of Vitronectin-Derived Adhesive Peptides

Leonardo Cassari [1], Annj Zamuner [2], Grazia M. L. Messina [3], Martina Marsotto [4], Hongyi Chen [5], Giovanni Gonella [6], Trevor Coward [6], Chiara Battocchio [4], Jie Huang [5], Giovanna Iucci [4], Giovanni Marletta [3], Lucy Di Silvio [6] and Monica Dettin [1,*]

[1] Department of Industrial Engineering, University of Padova, Via Marzolo 9, 35131 Padova, Italy; leonardo.cassari@phd.unipd.it
[2] Department of Civil, Environmental, and Architectural Engineering, University of Padova, Via Marzolo 9, 35131 Padova, Italy; annj.zamuner@unipd.it
[3] Laboratory for Molecular Surface and Nanotechnology (LAMSUN), Department of Chemical Sciences, University of Catania and CSGI, Viale A. Doria, 6, 95125 Catania, Italy; gml.messina@unict.it (G.M.L.M.); gmarletta@unict.it (G.M.)
[4] Department of Science, Roma Tre University, Via della Vasca Navale 79, 00146 Roma, Italy; martina.marsotto@uniroma3.it (M.M.); chiara.battocchio@uniroma3.it (C.B.); giovanna.iucci@uniroma3.it (G.I.)
[5] Department of Mechanical Engineering, University College London, London WC1E 6BT, UK; hongyi.chen.16@ucl.ac.uk (H.C.); jie.huang@ucl.ac.uk (J.H.)
[6] Faculty of Dentistry, Oral & Craniofacial Sciences, King's College London, London SE1 9RT, UK; giovanni.gonnella@kcl.ac.uk (G.G.); trevor.coward@kcl.ac.uk (T.C.); lucy.di_silvio@kcl.ac.uk (L.D.S.)
* Correspondence: monica.dettin@unipd.it; Tel.: +39-049-8275553

Table S1. XPS results.

Sample	Signal	Assignment	BE (eV)	FWHM	Internal Atomic ratios (%)	Atomic ratios
**PEEK**	C1s	C_{ar}, C-C	284.7	1.52	73	0.61
		C-N, C-O	286.3		23	0.19
		C=O	287.7		4	0.03
	O1s	C=O	531.4	1.79	51	0.08
		C-O	533.4		49	0.08
**PEEK-HVP**	C1s	C_{ar}, C-C	284.7	1.56	60	0.41
		C-N	286.0		25	0.17
		C-O	287.5		9	0.06
		C=O	288.7		6	0.04

	N1s	C=N	398.6	1.63	7	0.005
		C-N	399.8		82	0.07
		$-N^{+}$	401.2	.	11	0.009
	O1s	C=O	531.6	2.15	69	0.16
		C-O	533.2		31	0.07
**PEEK-D2HVP**	C1s	C_{ar}, C-C	284.7	1.52	75	0.60
		C-N	286.1		17	0.14
		C-O	287.5		5	0.04
		C=O	288.4		3	0.02
	N1s	C=N	398.2	1.58	10	0.007
		C-N	399.7		74	0.05
		$-N^{+}$	400.7	.	16	0.01
	O1s	C=O	531.4	2.11	72	0.095
		C-O	532.9		28	0.037

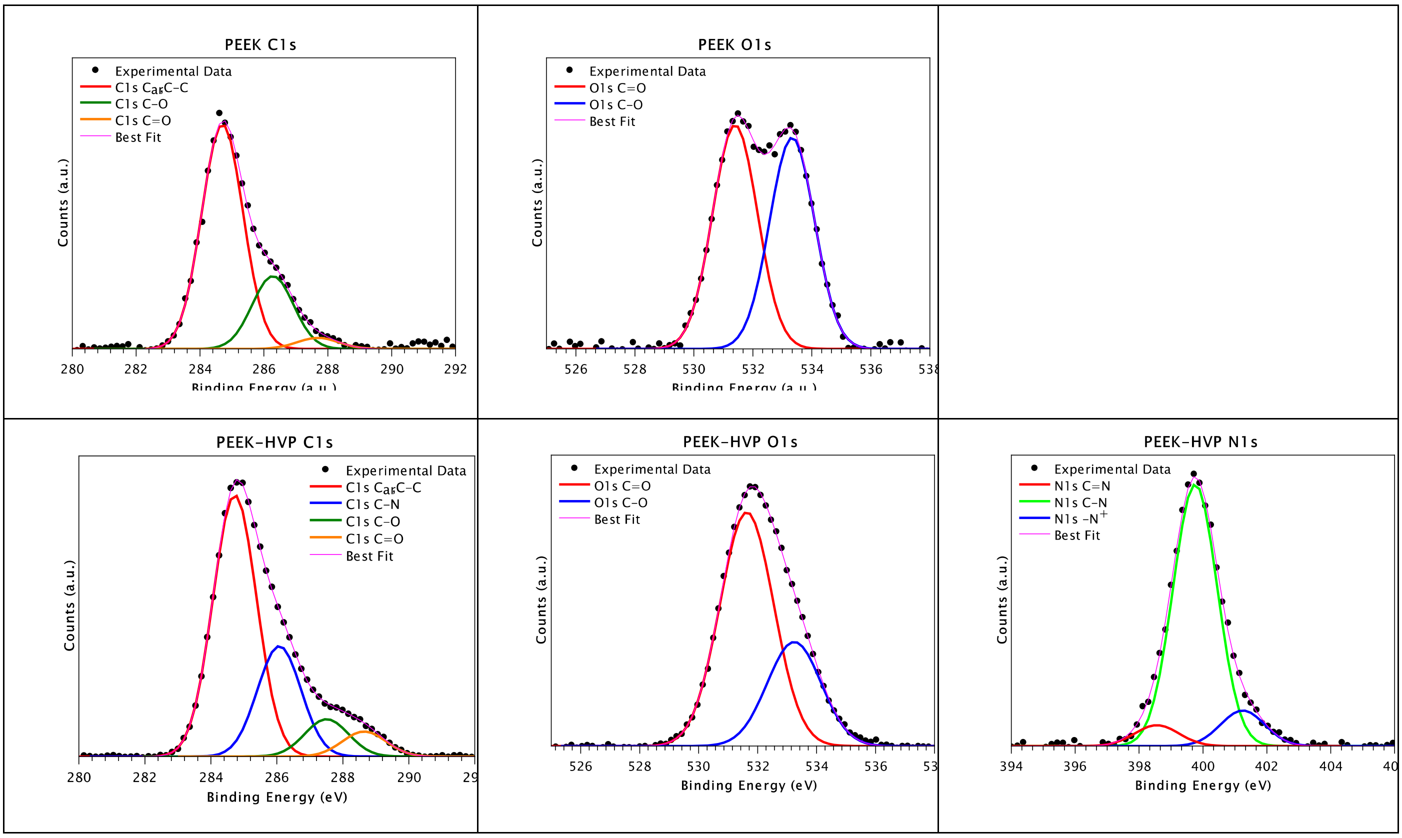

Figure S1. XPS spectra and peak fit results of sample PEEK and PEEK-HVP

Disclaimer/Publisher's Note: The statements, opinions and data contained in all publications are solely those of the individual author(s) and contributor(s) and not of MDPI and/or the editor(s). MDPI and/or the editor(s) disclaim responsibility for any injury to people or property resulting from any ideas, methods, instructions or products referred to in the content.
